# One-class support vector machines for detecting population drift in deployed machine learning medical diagnostics

**DOI:** 10.1038/s41598-025-94427-x

**Published:** 2025-04-09

**Authors:** William S. Jones, Daniel J. Farrow

**Affiliations:** Centre of Excellence for Data Science, Artificial Intelligence and Modelling (DAIM), Faculty of Science and Engineering, University of Hull, Hull, UK

**Keywords:** Population drift, Covariate shift, Machine learning, Medical diagnostics, Monitoring, Deployment, Scientific data, Statistics, Computational science, Software, Information technology, Breast cancer, Diagnosis

## Abstract

Machine learning (ML) models are increasingly being applied to diagnose and predict disease, but face technical challenges such as population drift, where the training and real-world deployed data distributions differ. This phenomenon can degrade model performance, risking incorrect diagnoses. Current detection methods are limited: not directly measuring population drift and often requiring ground truth labels for new patient data. Here, we propose using a one-class support vector machine (OCSVM) to detect population drift. We trained a OCSVM on the Wisconsin Breast Cancer dataset and tested its ability to detect population drift on simulated data. Simulated data was offset at 0.4 standard deviations of the minimum and maximum values of the *radius_mean* variable, at three noise levels: 5%, 10% and 30% of the standard deviation; 10,000 records per noise level. We hypothesised that increased noise would correlate with more OCSVM-detected inliers, indicating a sensitivity to population drift. As noise increased, more inliers were detected: 5% (27 inliers), 10% (486), and 30% (851). Therefore, this approach could effectively alert to population drift, supporting safe ML diagnostics adoption. Future research should explore OCSVM monitoring on real-world data, enhance model transparency, investigate complementary statistical and ML methods, and extend applications to other data types.

## Introduction

Artificial Intelligence (AI), machine learning (ML) medical diagnostics are tests that apply ML models to clinical data to help determine the presence, absence, extent of a disease; predict its likelihood; or prognosticate outcomes in a patient. They are increasingly being developed and used in clinical settings and are primed to revolutionise healthcare^1–5^.

One significant advancement driving this trend is that modern ML models are trained end-to-end to produce desired outputs, bypassing the laborious feature engineering step. Additionally, the accessibility of relatively low-cost computer hardware for model development, and the convenience of open-source ML model architectures and libraries (e.g. TensorFlow, PyTorch, Scikit-learn) make it possible to design, build and deploy (in the technical sense) ML models for medical diagnostic tasks with relative ease^2^. These models are typically trained on specific and narrow patient populations, whose characteristics closely match the population in which the test will be used in clinical practice^2^. This careful selection ensures that the model’s performance on the training data can effectively translate to the intended real-world clinical setting.

However, despite their potential, these models face several unresolved technical challenges, many relating to the way they are trained. One of the most significant challenges is the phenomenon known as ‘population drift,’ also referred to as ‘covariate shift’, ‘data drift’, ‘domain drift,’ and other terms. Terminology in this area is inconsistent^2,6–9^, but we consider population drift to the be most appropriate and intuitive in the context of medical research. Population drift occurs when the data distribution of input features (i.e. variables) changes between the training phase and real-world deployment. In other words, it occurs when the patient data used to train the model differs substantially from the patient data encountered when deployed in clinical practice^2,6,8,10,11^. For example, imagine you trained an ML model on a patient population of middle-aged men with controlled hypertension (characteristics X, Y, and Z). In regulatory terms, this information is captured by the ML model’s ‘intended use/purpose’. However, during deployment, the patients processed by the ML system may include middle-aged men with both controlled and uncontrolled hypertension (a subtle shift, e.g. X, Y, and W) or a broader population of older adults and women with varying comorbidities (a significant shift, e.g. A, B, and C). Both changes, and any change in population characteristics represent potential forms of population drift, which can affect the model’s performance and reliability in clinical practice. This concept can be expressed formally in several different ways. We conceptualise it as follows:

$$\:{P}_{\text{t}\text{r}\text{a}\text{i}\text{n}}\left(X\right)\ne\:{P}_{\text{d}\text{e}\text{p}\text{l}\text{o}\text{y}}\left(X\right)$$

$\:{P}_{\text{t}\text{r}\text{a}\text{i}\text{n}}\left(X\right)$ is the distribution of the input features in the training dataset. $\:{P}_{\text{d}\text{e}\text{p}\text{l}\text{o}\text{y}}\left(X\right)$ is the distribution of the input features in the deployment dataset. The inequality $\:{P}_{\text{t}\text{r}\text{a}\text{i}\text{n}}\left(X\right)\ne\:{P}_{\text{d}\text{e}\text{p}\text{l}\text{o}\text{y}}\left(X\right)$ characterises population drift; the scenario in which the distribution of the input features differs between training and deployment environments.

Population drift can occur for various reasons^2,3,6,7,10,12,13^, which stem from the non-stationary, dynamic nature of healthcare systems. Reasons include (1) the model being applied to a new demographic: used in a different clinical settings, or changes in standard of care, new treatments, seasonality variations, or the emergence of new diseases which disrupt pathways (e.g., COVID-19); (2) changes in technology: the introduction of new acquisition devices, medical tests, IT practices, software, or infrastructure, can alter the data on which the model relies; (3) changes in behaviour: shifts in clinical behaviour (e.g., new incentives that influence data collection, differential reimbursement), patient behaviour (e.g., increased diagnostic evaluations following a high-profile diagnosis or death) or clinical practices (e.g., new data management approaches, changes in care pathways can affect the model’s performance). The use of the ML model itself might induce behavioural changes in users, such as overreliance or automation bias^14^. ML models may also be vulnerable to adversarial attacks, where intentional perturbations are introduced into the input data to manipulate model outputs^15,16^.

Several studies have documented the adverse effects of population drift on ML models applied to real clinical data, across various disease and clinical areas^10,17–21^. When population drift occurs, it can significantly reduce the model’s performance, its diagnostic accuracy^2,5,8,10,13,14,19^, likely because the feature distributions have shifted to regions where the model performs poorly. Such degradation can result in incorrect diagnoses, with potentially life-threatening consequences for patients, and leading to various healthcare system inefficiencies. Therefore, it is imperative that ML models are monitored after deployment to detect shifts in data distributions. Moreover, regulatory bodies mandate the monitoring of AI systems for such issues^3,7,22^.

Population drift can be subtle and hard to detect. There are several proposed methods for monitoring ML models for population drift, but these are either methodologically suboptimal or not practical. The principal recommendation is to track the performance or accuracy of the ML model over time^2,23^, essentially performing a periodical or continuous clinical evaluation of the model against an appropriate benchmark/reference standard (i.e. that used to generate the ground truth labels during the training phase). This would be the ideal methodological approach, because the performance of the ML model is the crucial component in delivering reliable patient care, and changes in performance would highlight potential population drift, prompting model retraining or retirement.

However, this approach is impractical due to difficulties in obtaining ground truth labels because it would require tracking individual patients throughout their healthcare episode, which could span several years and may even necessitate additional tests beyond the standard of care. Monitoring performance through clinical evaluations would also be highly time consuming, labour intensive, and, perhaps most prohibitively, expensive^8,24^. It is often suggested that models be retrained using a new dataset obtained from the new deployment setting etc. as a means of mitigating population drift. While these methods could help the model adjust to new data distributions, the challenge is that it is unrealistic to assume that data will be readably available. Again, collecting new labelled data suffers from the impracticalities described above. There will also be regulatory challenges with this approach, since retrained models will themselves need regulatory approval, which is an arduous process.

It is more realistic, therefore, to try and identify population drift using unsupervised methods that do not rely on reference standard test and associated ground truth labels from new patients. One such approach was explored by Duckworth et al.^12^, who investigated the use of explainable ML techniques to monitor data distribution shifts, focusing on identifying high-risk patients in the emergency department; Explainable AI techniques aim to make ML models more interpretable by providing insights into how specific features influence model predictions. They trained a gradient-boosted decision trees (XGBoost) classifier on pre- and post-COVID-19 datasets and observed a drop in accuracy post-COVID. From their XGBoost model they derived SHapley Additive exPlanations (SHAP) values to quantify feature contributions. Differences in SHAP values indicated population drift. Therefore, Duckworth et al. suggested the tracking of SHAP values as a marker for potential population drift. However, a limitation of this approach is that the SHAP values serve only as surrogate markers for population drift, not direct measures. Additionally, subtle shifts may be harder to detect, and no clear threshold for population drift exists on this approach, making subjective interpretation necessary.

Similarly, Ackerman et al.^8^ (preprint), propose an unsupervised method that tracks a classifier’s confidence score to detect signs of population drift. Their approach uses the distribution of confidence values to identify when data has drifted from the training set distribution. However, like Duckworth et al., this method faces limitations. Confidence scores are not direct measures of population drift and may fail to detect subtle changes. And again, there is no clear threshold for the confidence values defining when a shift has occurred, so requiring subjective interpretation.

There is also a strong push for developing ML medical diagnostic models that are robust to population drift and can generalise to new, potentially out-of-distribution data. While this is an important area of research, it is not mutually exclusive with population drift detection; rather, the two approaches are complementary. To our knowledge, no ML medical diagnostic model has been developed that eliminates the possibility of population drift. Even if such a model were to exist, it would still be prudent to monitor for drift.

Other potential approaches include the application of statistical measures, such as the Kolmogorov-Smirnov (KS) test, Wasserstein distance, Kullback–Leibler (KL) Divergence, Local Lipschitz etc. These statistics can be used to determine the degree of difference between the distributions of the training and deployment data. These statistical measures are quantifiable and allow for visualisation of differences between individual variables, but suffer from similar issues described above, particularly, subjective determination of what constitutes population drift, and difficulty with interpretation of high-dimensional, multivariate datasets.

Unfortunately, none of these approaches offer a complete solution to population drift detection, though they may contribute to a broader strategy. What is missing is an unsupervised technique (not requiring ground truth labels from new patients using the deployed system) that more directly measures population drift, can be applied to new individual patient data, captures complex relationships in high-dimensional datasets, requires minimal subjectivity in determining drift, and is both easy to implement and gives understandable outputs to users. Such a method could serve as a foundation upon which the above-described approaches could be layered. Here, we propose the use of a one-class support vector machine (OCSVM) as this foundational model. Specifically, we suggest training an OCSVM on the original training dataset of an ML medical diagnostic system. The OCSVM would then be used to detect population drift in new patients processed by the deployed diagnostic system. The use of OCSVMs is established in applications of novelty detection^25–30^, and so is sufficiently motivated for exploration in this domain.

In this study, we describe and evaluate the use of an OCSVM to detect population drift in a hypothetically deployed ML medical diagnostic. Real-world data on population drift is difficult to acquire, as this phenomenon has only recently come into focus due to the growing prominence of AI and ML models. Therefore, we simulate population drift using the well-known and publicly available Wisconsin Breast Cancer (Diagnostic) Dataset^31,32^. This dataset enables a clear proof-of-concept demonstration and allows for reproducibility and extension of results.

## Methods

### Study design

We trained a one-class support vector machine (OCSVM) on the Wisconsin Breast Cancer (Diagnostic) dataset^31^; hereafter abbreviated to WBC dataset. The OCSVM model was developed in Python (v3.9.13) via the scikit-learn library (v1.0.2)^33^ using the OneClassSVM class.

### Dataset and preprocessing

The WBC dataset consists of data derived from fine needle aspiration (FNA) of breast masses. Features are computed from digitised images of FNA samples, capturing various geometric characteristics/properties of the cell nuclei present in the images, detailed below. Each sample is labelled as either benign or malignant, making the dataset ideal for binary classification tasks in medical diagnosis; See Wolberg, W., et al.^31^ for further details FNA process and the methodology used to compute these features.

The WBC dataset has 569 instances (i.e. patient records) where each instance corresponds to a breast tumour sample. The dataset has 30 numerical/continuous input features (i.e. independent variables) and one target feature (i.e. the dependent variable or class label) ‘diagnosis’, a binary variable with two possible outcomes: benign (*n* = 357, encoded as ‘B’) and malignant (*n* = 212, encoded as ‘M’). The dataset contains no missing values. As indicated above, the input features describe characteristics of the tumour cell nuclei, computed from a digitised image of a FNA. 

For preprocessing, we performed multicollinearity analysis via Pearson correlation coefficients between all feature pairs. Features exhibiting a high degree of correlation, defined by an absolute |r| > 0.9, were considered redundant and subsequently removed to reduce multicollinearity. Practically, this resulted in the removal of the following input features: *perimeter_mean*,* area_mean*,* radius_worst*,* perimeter_worst*,* area_worst*,* texture_worse*,* concave points_mean*,* perimeter_se*,* area_se*.

To ensure that both benign and malignant classes are equally represented in the feature space of the OCSVM, and to mirror processes that would occur in the development of a ML medical diagnostic, we applied synthetic minority oversampling technique (SMOTE)^34^. This balanced the dataset by upsampling the malignant class from 212 to 357 instances, resulting in 357 benign and 357 malignant samples, for a total of 714 instances.

Following the application of SMOTE, we removed the ‘diagnosis’ target feature, since this is not required and cannot be used in OCSVM, an unsupervised technique that focuses on the input features only. Next, we scaled the data, which standardises features by removing the mean and scaling to unit variance, resulting in a mean of 0 and standard deviation of 1. Scaling is performed to ensure that all features contribute equally to the model, preventing features with larger numerical ranges from disproportionately influencing the OCSVM’s performance. Scaling is also a common step in developing ML medical diagnostics. At this stage, the WBC dataset is prepared for use in the OCSVM model, consisting of 714 instances and 21 continuous, scaled input features.

### OCSVM model

The OCSVM was introduced by Schölkopf et al. in 2001^35^. It is an unsupervised learning algorithm primarily used for novelty or outlier detection, where the goal is to determine whether a new instance, observation, record (synonyms) deviates significantly from the distribution of the training data.

Consider a dataset with $\:n$ observations, each characterised by $\:p$ features. Novelty detection addresses the question of whether a new observation is sufficiently similar to the existing $\:n$ observations to be considered part of the same distribution. The OCSVM learns a boundary, or frontier, delimiting the contour of the initial observations’ distribution, plotted in $\:p$-dimensional space. If subsequent observations fall within the frontier they are classified as inliers, indicating they are from the same population as the training data. Conversely, if new observations fall outside the frontier, they are classified as novelty or outliers, indicating they are not from the same population. When a new observation is classified as an outlier, an individual example of population drift has potentially occurred. In this case, one must be cautious running this observation through the associated ML medical diagnostic trained on this dataset, because the performance/accuracy values might be incorrect. This one example of population drift could also indicate a more systemic problem.

In this study, we fit the OCSVM to the WBC dataset. The model was configured with a radial basis function (RBF) kernel, automatic gamma scaling, and contamination parameter nu (or $\:v$) set to 0.01 (see Table 1 for summary of parameters). The RBF kernel defines the shape of the boundary that separates the inlier data points, from the outliers. The RBF kernel is well-suited for capturing non-linear relationships in complex datasets. It allows the model to create flexible, curved boundaries around the data. Gamma scaling controls how much influence each data point has on the boundary; a higher gamma makes the boundary very sensitive to data points, whereas lower gamma creates a less sensitive, smoother boundary. Setting this to automatic ensures that the influence is scaled to the number of features in the dataset: 1/$\:p$ features. This simplifies the model tuning, and potentially reduces overfitting. The contamination parameter nu determines the fraction of data points that the model should treat as outliers; nu = 0.01 assumes that 1% of the data might be outliers.

**Table 1:** Configuration parameters of the OCSVM model used in this study for the WBC dataset.

Parameter	Value
Kernal	Radial basis function (RBF)
Gamma scaling	Automatic
Contamination ($\:v$)	0.01

The proposal in this study is that the OCSVM model trained on the WBC dataset can serve as a mechanism to detect population drift in new observations that are processed through ML medical diagnostic models developed on the same dataset. The OCSVM learns a boundary based on the distributions of the WBC data’s features.

For example, consider an ML model trained to classify patients as having benign or malignant tumours using the same WBC data. When deployed, new patient data can first be passed through the OCSVM to determine whether the instance is an inlier or outlier when compared to the training data. If classified as an outlier, this suggests the new patient’s data deviates from the original training distribution, indicating potential population drift. In such cases, caution is warranted with the subsequent application of the ML diagnostic model, as the patient’s data may not align with the training data distribution. Conversely, if the OCSVM classifies the new instance as an inlier, it suggests the data is consistent with the training data distribution, allowing the ML classifier to be used with a greater degree of confidence.

### Data simulations and model evaluation

To evaluate the OCSVM model fitted to the WBC dataset, we simulated drifted populations centred in two groups. To select central locations for these outlier populations, we chose the real data rows with the maximum (and minimum) values of *radius_mean* and added (subtracted) 0.4 standard deviations to (from) that feature. The feature *radius_mean* plays a significant role in predicting tumour classification in the WBC dataset^36^, and so was a sensible variable to manipulate in the simulations. The choice of these values was intended to simulate a plausible and clinically meaningful form of population drift caused by changes in the patient population. Higher values represent a potential shift towards later-stage patients with likely malignant tumours, while lower values simulate a shift towards early-stage patients entering the diagnostic pathway, with likely benign tumours. Gaussian noise was added to all feature values, to model a spread in the outlier population. We adopted three noise levels, 5%, 10% and 30% of the standard deviation of the real population. For each noise level, 10,000 records were simulated (5,000 positioned 0.4 standard deviations below the minimum and 5,000 positioned 0.4 standard deviations above the maximum *radius_mean* value*)*; 30,000 total. The noise levels and 0.4 standard deviations offsets were chosen to demonstrate the concept and include some overlap between the outliers and the original population.

The simulated records were then processed through the trained OCSVM model to observe how they would be classified. It was predicted that as noise levels increased, the model would identify more records as inliers, as a more dispersed group of outliers would have a larger number of instances landing in the inlier boundary. This trend would support the OCSVM’s expected sensitivity to change in population characteristics. By evaluating the proportion of inliers and outliers detected at each noise level, we assessed the model’s capacity to recognise these synthetic shifts in patient population characteristics.

**Fig. 1:**
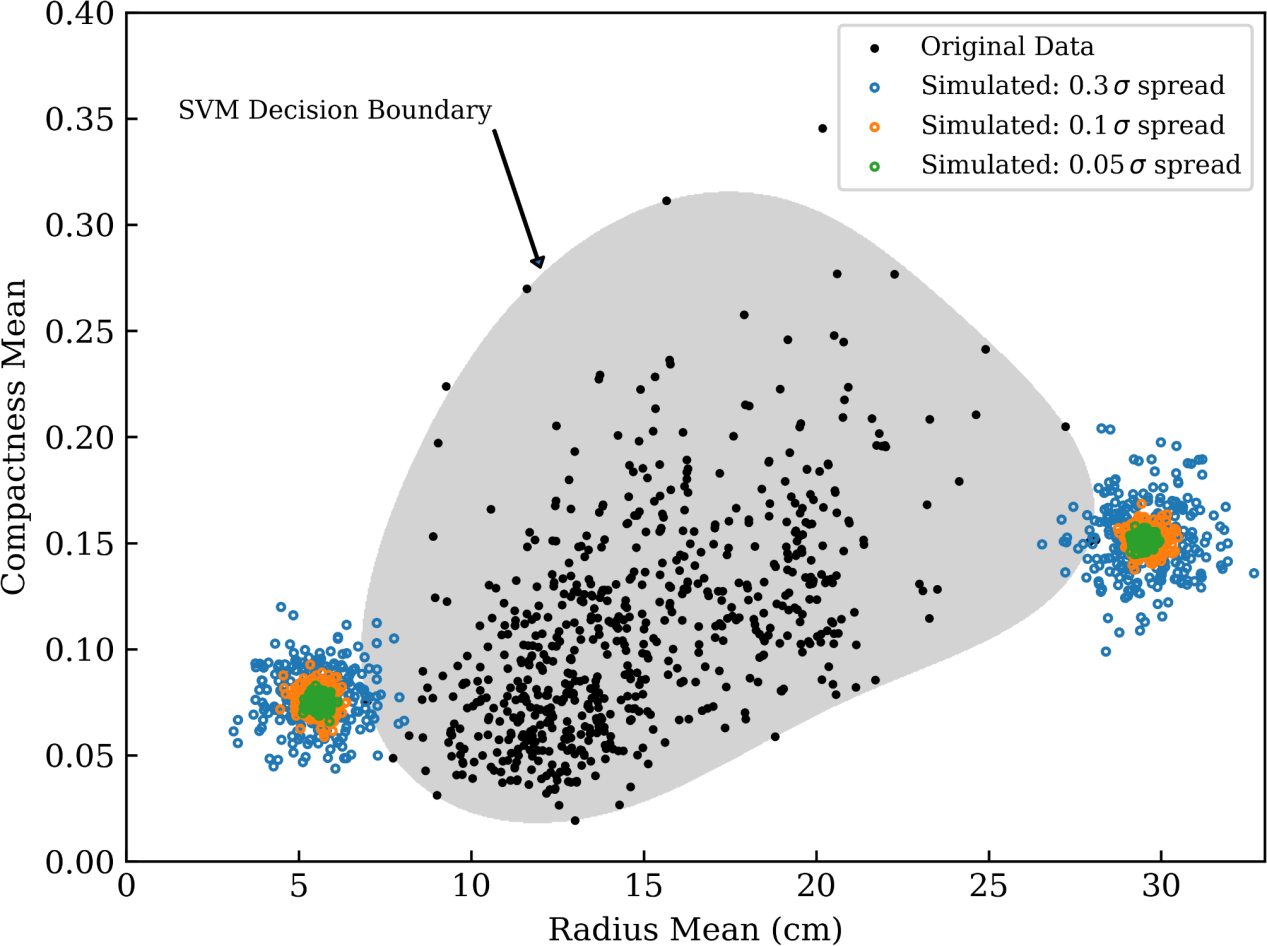
Decision boundary of a one-class support vector machine model trained on the Wisconsin Breast Cancer dataset. Exclusively for this visualisation, we trained a OCSVM on only two variables: radius_mean and compactness_mean, to allow for interpretable visual representation, as (+ 3) higher-dimensional data cannot be directly visualised. Simulated test data at three noise levels (5%, 10%, and 30% of the standard deviation) are shown in green, orange and blue, respectively, with outlier groups positioned 0.4 standard deviations below the minimum and above the maximum radius_mean values in the dataset. As noise increases, more simulated outliers are classified as inliers, demonstrating a sensitivity to population drift. Only a subset of the simulations are presented in this figure.

Finally, to provide an intuitive understanding of how the OCSVM monitors and classifies drift, we trained a model using two variables: *radius_mean* and *compactness_mean*. Limiting the model to two variables allows for an interpretable visual representation of the OCSVM in action, as data with more than three variables cannot be directly visualised. To further illustrate its functionality, we simulated test data at three noise levels (5%, 10%, and 30% of the standard deviation), with outlier groups positioned 0.4 standard deviations below the minimum and above the maximum *radius_mean* values in the dataset (see Fig. 1 in Results).

## Results

The OCSVM model’s performance was evaluated on simulated test data at three noise levels (5%, 10%, and 30% of the standard deviation of the real population) positioned 0.4 standard deviations below the minimum and 0.4 standard deviations above the maximum *radius_mean* values, to assess its ability to detect population drift.

**Table 2:** Classification of simulated outliers as inliers by the OCSVM model at increasing noise levels, showing sensitivity to population drift decreases as the overlap between the shifted and original populations increases.

Noise level (%)	Records classified as inliers (total records per level = 10,000)	Proportion classified as inliers (%)
5	27	0.27
10	486	4.86
30	851	8.51

At the lowest noise level (5%), the model classified 27 records as inliers (27 out of 10,000). As noise increased, the proportion of records classified as inliers increased, with 486 outliers at 10% noise and 851 at 30% noise; see Table 2 for summary. This trend aligns with our prediction that the model’s false negative rate would increase as the simulated data increased its overlap with the original training distribution, reflecting the model’s sensitivity to incremental population drift. We also note that these results will be sensitive to how far the centre of the outlier population will be, with more subtle shifts being harder to detect.

## Discussion

In this study, we explored the use of a one-class support vector machine (OCSVM) for detecting population drift in deployed ML medical diagnostics. We fit a OCSVM to the Wisconsin Breast Cancer (WBC) Diagnostic dataset, and simulated data with varying degrees of noise to evaluate the OCSVM’s performance.

The simulation results indicate that the OCSVM model is sensitive to population drift. Consequently, we believe this analysis demonstrates proof of concept for the OCSVM’s potential to detect and monitor subtle population drifts, though further work is required to fully assess its efficacy.

Future research should evaluate the use of OCSVMs in real-world clinical settings and datasets to determine their performance and general utility. Additionally, it is important to explore other one-class classifiers (e.g. Isolation Forests, Robust Covariate, …), and complementary statistical and ML approaches to see how they could be integrated for improved performance. There may also be a deep Bayesian solution to detecting population drift that could also form part of a continuous learning model. Dimensionality reduction techniques were not used in this study and should be explored to assess their impact on OCSVM performance. In addition, our modelling of outlier populations in this proof-of-concept work was limited. Future research could focus on more sophisticated modelling of the outlier population, which should include more subtle shifts in the centres of the outlier populations and correct modelling of the covariance between features. Moreover, the output from the OCSVM is binary, classifying data points as either inliers or outliers. But we know that dichotomisation discards potentially valuable information and oversimplifies the complexity of data^37,38^. Future research should explore approaches that provide continuous output values, while simultaneously addressing the challenge of interpreting these outputs in a clinically meaningful way.

Follow-up work should also focus on methods for tracking and predicting population drift prospectively, identifying potential vulnerabilities before they affect model performance^3^. Consideration should also be given to the regulatory implications of population drift, including gaining the perspectives of regulators. WJ conducted a rapid scoping review to identify relevant literature on this topic. While this approach was appropriate given the urgency and nascent nature of the research, a full systematic review of the literature would potentially allow for a deeper comparative and quantitative analyses of proposed solutions, particular as more work is produced in this area. Finally, the issue of population drift extends beyond ML medical diagnostics, and research should explore whether the outlined approach can be applied in other domains.

There are several limitations and uncertainties with the OCSVM approach to detecting population drift, with a major concern being the model’s limited transparency and explainability in its decision-making process. Although boundaries and support vectors are mathematically defined, pinpointing the exact feature or combination of features driving each classification remains challenging. Some recent work has begun exploring the integration of explainable AI techniques with OCSVMs to address this gap^39^, and further investigation in this area is required.

It is also not clear whether this OCSVM approach would be effective in identifying population drift in ML medical imaging datasets and models, and further work is necessary to explore this. However, there is no immediate reason to rule out its potential efficacy, particularly when applied after feature extraction processes, e.g. post convolutional filters in convolutional neural networks. In addition to OCSVM, alternative methods could be explored for detecting population drift in medical images. For example, Mahalanobis distance has been proposed for identifying shifts in feature space^40^, while autoencoders and classifiers can be used to detect deviations from the expected image patterns^41^. Cosine distances between feature vectors also offer a promising approach for measuring shifts in distribution^42^. These methods, including those mentioned earlier, alongside OCSVMs, could form a robust toolkit for monitoring and detecting population drift across various data modalities, including medical imaging.

## Conclusion

In conclusion, we believe this study constitutes proof of concept for the use of a one-class support vector machine (OCSVM) in detecting population drift within deployed machine learning medical diagnostics. The model demonstrated sensitivity to subtle population drift indicating its potential for monitoring changes in data distributions. This model is also capable of real-time detection of population drift from individual patients, without ground truth labels / reference standard tests of the patient’s outcome. Follow-up work should explore OCSVM monitoring on real-world data, enhance model transparency, investigate complementary statistical and ML methods, address regulatory issues, and extend applications to other data types, such as medical images. By addressing these areas, the OCSVM approach could serve as a foundational and efficacious tool for detecting population drift in machine learning medical diagnostics and other important domains.

## Data Availability

The Wisconsin Breast Cancer (Diagnostic) dataset is publicly available from multiple sources, including: https://archive.ics.uci.edu/dataset/17/breast+cancer+wisconsin+diagnostic. The model and simulation Python code can be made available on reasonable request to the corresponding author.
